# Is ostension any more than attention?

**DOI:** 10.1038/srep05304

**Published:** 2014-06-16

**Authors:** Joanna Szufnarowska, Katharina J. Rohlfing, Christine Fawcett, Gustaf Gredebäck

**Affiliations:** 1 Emergentist Semantics Group, CITEC, Bielefeld University, Germany; 2 Department of Psychology, Uppsala University, Sweden

## Abstract

According to natural pedagogy theory, infants are sensitive to particular ostensive cues that communicate to them that they are being addressed and that they can expect to learn referential information. We demonstrate that 6-month-old infants follow others' gaze direction in situations that are highly attention-grabbing. This occurs irrespective of whether these situations include communicative intent and ostensive cues (a model looks directly into the child's eyes prior to shifting gaze to an object) or not (a model shivers while looking down prior to shifting gaze to an object). In contrast, in less attention-grabbing contexts in which the model simply looks down prior to shifting gaze to an object, no effect is found. These findings demonstrate that one of the central pillars of natural pedagogy is false. Sensitivity to gaze following in infancy is not restricted to contexts in which ostensive cues are conveyed.

Ostensive signals are understood as cues designed by a communicator to generate an interpretation of communicative intention in an addressee^1^. According to Csibra and Gergely's natural pedagogy theory^2^, this certain class of social stimuli has a special meaning to young infants. That is, infants are sensitive to particular ostensive cues that communicate to them that they are being addressed and that they can expect to learn referential information. These ostensive cues, to which infants are particularly responsive, are proposed to be restricted to direct gaze, infant directed speech, and/or contingent responsivity^1^. The most prominent example of this putative process is that gaze following is demonstrated by 6-month-old infants only if gaze is preceded by ostensive cues such as direct gaze or infant directed greeting, and not when these cues are replaced with a non-ostensive and non-social animation^3^. From such findings, it is concluded that ostensive cues are what allow gaze following and other instances of referential learning to occur in infancy^2^.

We argue that these claims are premature. It is possible that infants' general attention to social behaviors increases covert attention to actors and to events that follow. Thus gaze following should be prominent after any social cue that heightens attention to the actor and his/her subsequent referential actions, including both ostensive cues and other attention-grabbing actions that do not conventionally serve a communicative purpose. In summary, the central divider between these two theories, natural pedagogy and attention modulation, is whether ostensive cues hold a unique contract on heightened responsiveness.

Several researchers have demonstrated infants' early sensitivity to an actor's head and eye movement that can lead to gaze following. Farroni, Johnson, Brockbank, and Simion^4^ claimed that four- to five-month olds' attention can be cued by the perceived model's motion (either her eyes or her head) toward a target rather than by the final direction of eye gaze. However, these experiments included a direct gaze toward the infant by the actor before the movement, a possible “ostensive cue” that could have contributed to the gaze following. Similarly, Moore, Angelopoulos, and Bennett^5^ claimed that the movement associated with gaze reorientation, in contrast to a static orientation toward the side, is crucial in establishing gaze following in infants. However before the experimenter turned his head and eyes toward an object, he engaged in a face-to face interaction with the infant, another potential “ostensive cue”. Thus, neither of these previous studies can tease apart whether movement cues alone can elicit gaze following without the presence of direct eye gaze to the infant.

In the current eye tracking study, we set out to test the claims of Csibra and Gergely^2^ – that ostensive cues hold a unique contract on infants' gaze following – with the addition of new critical control conditions that were missing in the original study of ostensive cues^3^. We compared six-month-old infants' gaze following under five different conditions that varied in whether the adult provided ostensive cues as in previous studies (Direct gaze); attention-grabbing, but non-ostensive cues (Shivering); potentially ostensive cues (Nodding or Nodding with direct gaze); or no cues at all (see Fig. 1 for selected frames from the stimuli). In line with previous results^3^, we expected more gaze following after direct gaze than no cues. However, critical to the current investigation is infants' gaze following tendencies during the Shivering condition. According to natural pedagogy, six-month-olds should not follow gaze in this condition whereas the attention modulation theory suggests that infants should follow gaze in response to this attention-grabbing, but non-ostensive cue. The two nodding conditions represent other situations with possible communicative intent as motivated by mothers' natural behavior towards a child^6^, and were included to enhance the variability and ecological validity of the stimulus material, providing data on the range of contexts in which gaze following can occur.

## Results

In order to examine infants' gaze following, the first gaze shift from the actor's head to an object after the actor started to turn toward one of two objects was recorded (beginning from the first frame of the actor's head turn, as in^3^). Specifically, the first head-to-object gaze shift for a given trial was coded as soon as the infant made a gaze shift from the Area of Interest including the model's head (*AOI head*) to one of the two Areas of Interest for the toys placed on the table (*AOI left toy* or *AOI right toy*) (see Fig. 2). This first gaze shift was coded as congruent if it was to the same side as the actor's head turn and incongruent if it was to the other side. Subsequently, the data for the four trials in each condition were aggregated to average differences scores (DS) between the number of trials with first fixation on the attended object (congruent gaze shift) vs. the unattended object (incongruent gaze shift) according to the following formula (see also^3^):


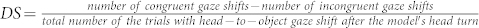


Gaze following was defined as a difference score that significantly exceeds zero (one-tailed single-sample t-tests, *p* < .05).

Replicating the original findings by Senju and Csibra^3^, infants followed gaze in response to Direct gaze (*t*(21) = 2.156, *p* = .022, *d* = 0.94,) and not in response to No cues (*t*(21) = −1.213, *p* = .199, *d* = 0.53). Critically, infants also followed gaze in the non-ostensive Shivering condition (*t*(21) = 2.045, *p* = .027, *d* = 0.89). The two conditions with potential ostensive cues (Nodding and Nodding with direct gaze) resulted in marginally significant gaze following and medium effect sizes (*t*(21) = 1.715, *p* = .051, *d* = 0.75, *t*(21) = 1.699, *p* = .052, *d* = 0.74, respectively). Additional analyses revealed significantly more gaze following for every action compared to No cues (one-tailed paired-sample t-tests, *p* < .05; Direct gaze: *t*(21) = −2.310, *p* = .016, *d* = 1.01; Shivering: *t*(21) = −2.030, *p* = .028, *d* = 0.89; Nodding: *t*(21) = −1.866, *p* = .038, *d* = 0.81; Nodding with direct gaze: *t*(21) = −1.764, *p* = .046, *d* = 0.77). Figure 3 presents the above-described results.

In addition to the difference score for the first head-to-object gaze shift, which is the standard assessment of infants' gaze following (e.g.^3^,^7^, two additional measurements were taken into account after the start of the actor's turn toward an object: the frequency of head-to-object gaze shifts and the duration of object fixation in the gazing phase (as in^3^. The results from these two further measurements are available online in the Supplementary Results section.

Attention (total fixation duration) to the model's head during her action was lower in the No cues condition (1196 ms 95% CI = +/− 202 ms) than each of the other conditions, which did not differ from each other (Direct gaze: 1458 ms 95% CI = +/− 214 ms; Shivering: 1501 ms 95% CI = +/− 201 ms; Nodding: 1503 ms 95% CI = +/− 201 ms; Nodding with direct gaze: 1566 ms 95% CI = +/− 211 ms).

## Discussion

Together, the current findings on infants' gaze following clearly demonstrate that one of the central pillars of natural pedagogy is false. Sensitivity to gaze following in infancy is not restricted to contexts in which ostensive cues are conveyed. The results from both gaze following and attention to the model are instead consistent with the attention modulation account, claiming that the previously demonstrated effect of ostension on gaze following is simply based on social attention mechanisms and that young infants' attention is high in response to attention-grabbing human actions, irrespective of conveyed communicative intent. Thus, irrespective of whether particular actions contained single attention-grabbing cue (Shivering), single ostensive cue (Direct gaze), potential ostensive cue (Nodding), or a combination of them (Nodding with direct gaze), they all redirected the infants' gaze towards a referent in contrast to No cues condition. The findings are also in line with a recent study demonstrating gaze following based on non-ostensive cues in toddlers^8^. In sum, while the current findings do validate prior reports demonstrating that direct gaze enhances gaze following, they put into question the putative role of natural pedagogy in explaining these results.

Our results are in line with other research suggesting that attention-based mechanisms can account for infants' acquisition of gaze-following^9^,^10^,^11^. The computational model of the emergence of gaze following skills in infant-caregiver interactions^9^,^10^ is based on the idea that “infants learn gaze following because they discover that monitoring their caregiver's direction of gaze allows them to predict where interesting visual sights occur”^9^ (p. 128). Thus in this framework it is the infant's prior interactions that are crucial for the infant to form an expectation about the adult's gaze direction being linked to a target object. The findings from our study – particularly the No cues condition which did not result in gaze following – add an additional caveat to this proposal, in that head movement and gaze to an object are not enough for infants to follow gaze. Thus even if infants have learned to follow gaze based on previous observations of head and eye movements to interesting sights, they still additionally require an attention-grabbing cue to do so. However, we have to highlight the fact that in contrast to research presented in^9^ and^10^, our experiments took place in laboratory conditions, in which the actors did not establish any natural interaction with children. It is thus possible that in such controlled conditions and without any history of interaction, infants need more cues to respond in a gaze following situation.

Future research can work to clarify whether there are gradations of infants' responsivity toward conventionalized ostensive cues and other salient social, and even non-social, stimuli. The results from the current and the original^3^ study, could suggest that a social stimulus (conventionalized or not) is necessary because infants did not follow the gaze when the actor's face was covered using a non-social animation^3^, whereas they did when social attention-grabbing cues were presented. However, it is also possible that the non-social animation could actually have functioned as a distractor in two ways: First, in the attention-getting phase, the animation could draw infants' attention to itself, rather than toward the actor since it was placed over her face. Second, once the animation disappeared, instead of following the gaze of the model, infants could have been confused because of the sudden absence of the attractive stimulus. A possible remedy would be to highlight the actor in a non-social way, for example by briefly shining a spotlight onto her face before she gazes at an object. Relatedly, Axelsson, Churchley and Horst^12^ have recently shown that a non-social cue such as illuminating a target object can sometimes be more helpful for preschoolers' word learning than a social pragmatic cue such as pointing. They propose that illuminating the target, in contrast to pointing to it, drew children's attention to the novel object more strongly and therefore helped them to encode and retain the novel name-object association. The authors use the term “ostensive naming” in reference to illuminating the target suggesting that helpful cues do not necessarily need to be social but rather they must simply be clearly demonstrative. However, in this study the illumination was applied in combination with direct eye gaze towards the children. Therefore it is likely that the set of cues rather than illumination alone was responsible for the learning effects. The need to tease apart whether a purely non-social attention-grabbing cue can lead infants to follow gaze or even learn words thus still remains.

In sum, the current findings demonstrate that gaze following is not limited to situations that include a particular set of ostensive cues as defined within Natural Pedagogy, but rather that attention plays an important role in eliciting gaze following. Future work should continue to explore the features of cues that infants respond to in order to determine the nature of their attention and the situations in which they are likely to learn from others. This might result in a redefinition of ostension, away from particular cues used to generate an interpretation of communication in the addressee and towards more general social attention mechanisms that a communicator can rely on.

## Methods

### Participants

Twenty-two six-month-olds *(M* = 6 months 12 days, *SD* = 10 days; 14 girls) participated in the experiment. Three additional infants were excluded from the final analyses because of inattentiveness (they shifted gaze from the actor's head to one of the objects after the actor's head turn in less than half of all trials). The study was approved by the regional ethical committee and written parental consent was obtained for all infants. Parents received a gift voucher worth approximately 10 euros for their participation.

### Stimuli

The stimuli consisted of a series of video clips with a head and shoulders view of a female actor seated at a table. Two colorful objects were placed on the table to either side of the actor, equidistant from her. The distance between the centers of the objects was 30 cm and the distance between the center of each object and the edge of the table was 15 cm. Each video began with the actor looking downward for 2 seconds. During this period, 1.3 seconds from the beginning of the video, a short ‘beep' signal (0.7 s) was heard. Then the actor raised her head slightly (approx. 20 degrees) but maintained downward gaze (1 s). Afterwards the actor performed one of five actions: Direct gaze (moving gaze up to look at the infant), Shivering (small rapid horizontal head movements, with approx. 25 degree angle and with downward gaze), Nodding (two consecutive slow vertical head movements, with approx. 40 degree angle and with downward gaze), Nodding with direct gaze (first direct gaze then nodding, also with approx. 40 degree angle, while maintaining direct gaze), or No cues (no additional movement and downward gaze). Each presented action lasted approximately 2 seconds. After each action, the actor moved her head and gaze to one side (within 1 s, with approx. 45 degree angle) and maintained it on the target object for 5 seconds (object and side fixated were randomized across trials).

**Figure 1:**
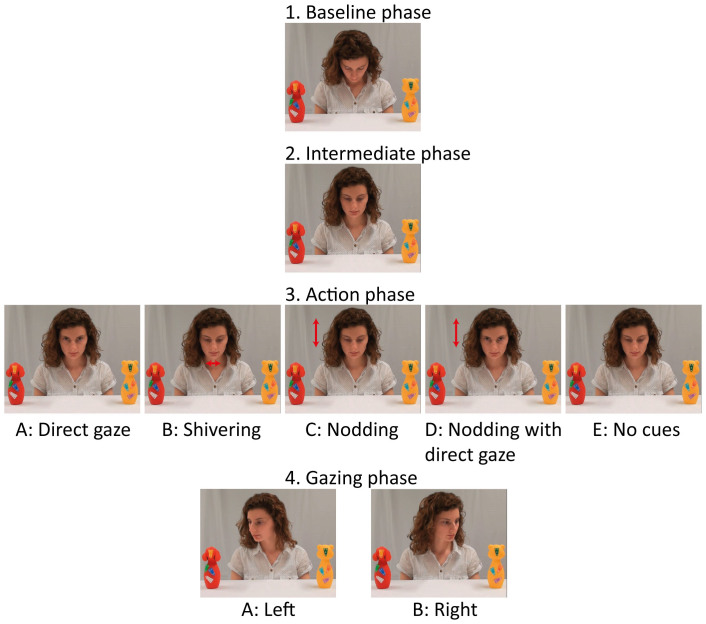
Selected frames from each phase of the stimuli. Each video clip started with (1) Baseline phase, followed by (2) Intermediate phase, (3) Action phase and (4) Gazing phase. The phases (1) and (2) were the same in all videos. In the 3rd phase, the model performed one of five types of actions (conditions): A, B, C, D or E; next, in the 4th phase, the model looked to one of the objects: either on the left or on the right side of the screen.

**Figure 2:**
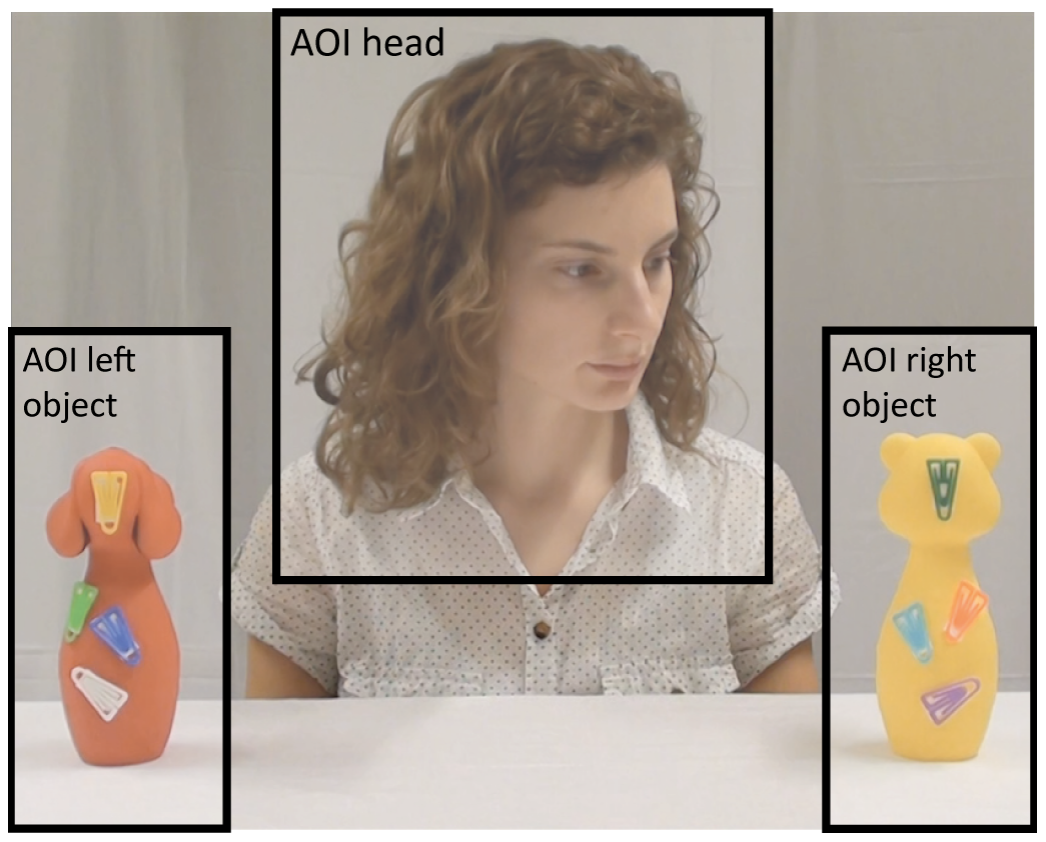
The defined areas of interest (AOIs). The coding of infant's gaze shifts started in the gazing phase (after the model started to turn toward one of the toys). The head-to-object gaze shift was coded as soon as the infant made a gaze shift from the *AOI head* to either the *AOI left object* or to the *AOI right object.* The AOIs in the video clips with other actors had analogous positions.

**Figure 3:**
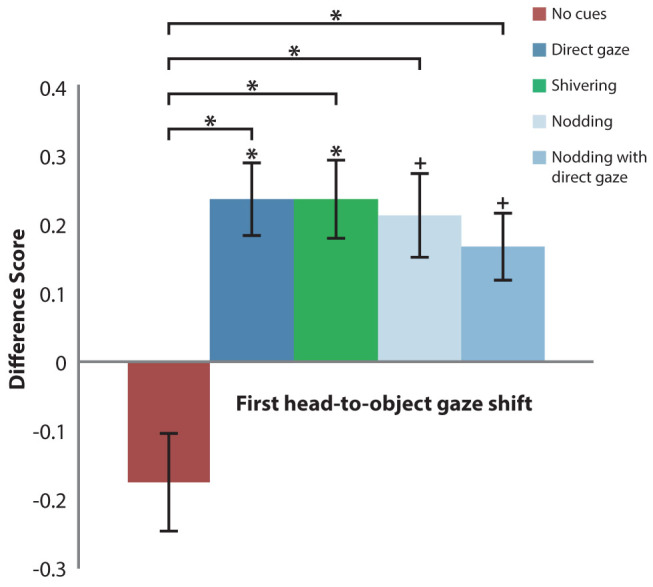
Measures of gaze following for No cues, Direct gaze, Shivering, Nodding and Nodding with direct gaze. The results from the main measurement (First head-to-object gaze shift) indicate that infants did not follow gaze in response to No cues but did in both ostensive Direct gaze and non-ostensive Shivering condition (*ps* < .05). Infants also appeared to follow gaze in the two conditions with potential ostensive cues: Nodding and Nodding with Direct gaze (*ps* = .05). Furthermore, paired-sample t-tests revealed more gaze following for every action compared to No cues (all *ps* < .05). Error bars represent SE; stars indicate the p-values < .05; pluses indicate the p-values = .05. P-values above the columns are based on single-sample t-tests against a DS of zero; p-values above the horizontal square brackets stem from paired-sample t-tests and depict the comparisons between No cues and each other condition.

In sum, the video clips had five possible *actions* that the actor performed before moving her head and gaze to one side to create the five conditions (Direct gaze, Shivering, Nodding, Nodding with direct gaze, or No cues). Additionally, each video clip ended with gaze to one of two *directions*: the actor looked at an object either on the left or right side of the screen (see Fig. 1 for selected frames from each phase of the presented video clips). Each of these ten videos was recorded with five different *actors* and each actor was presented with a unique pair of toys. In total, the corpus of 50 video clips was recorded such that each of five *actors* performed each of five types of *actions* with gaze to each of two *directions* (see Supplementary Fig. S1 online for a schematic diagram of the corpus of prepared video clips). The first part of each actor's activity (looking downward for 2 seconds) and the last one (looking at a toy for 5 seconds) lasted for the same amount of time, in each of 50 video clips. The duration of performing each of the three remaining activities (raising the head, performing one of five actions and moving the head and gaze toward one of two toys) varied slightly across the recordings and actors. This variation was allowed to retain the naturalness of the movements. However, before the recordings of the video clips, all actors were precisely trained to perform each part of the presentation in possibly the same way and in the same speed, so these differences are negligible.

From this corpus of 50 video clips, five clip sequences (i.e., five versions of the stimuli) were created. Each video clip was one trial. Each version consisted of four blocks of five trials each. In a particular block, each trial presented a different condition (i.e., action). Each condition within a given block was presented by a different actor. Within a particular version, the same actor presented a given condition only once. The order of the conditions within a block and the order of the actors were randomized across blocks. Furthermore, the direction in which the actor looked was quasi-randomized with never more than two looks in a row to the same direction. Before the start of each trial, a brief animation with sound (attention-getter) was presented on the screen. After every block of five trials, a longer colorful animated movie with sound was presented for 15 seconds. The order of the stimuli as they were arranged in one particular version of the experiment can be found as Supplementary Figure S2 online. Each version presented the same type of stimuli (four trials of each condition) but differed in the order of the conditions across the blocks and the video clips used. By creating five versions of the stimuli we aimed to exclude the risk of infants' preference of a specific condition because of its primary presentation in a particular block.

### Procedure

Infants were seated on their parent's lap approximately 60 cm from the screen of a Tobii T120 remote eye tracker. This eye tracker has a reported accuracy of 0.5 visual degrees and freedom of head movement within 30 × 22 × 30 cm. Gaze was recorded at 60 Hz. After a 5-point calibration procedure, infants watched the first two blocks of trials (10 trials total). Infants then had a break of approximately 7 minutes during which they could play with their parent. After the break, infants were seated again on their parent's lap, a new calibration was performed, and they watched the remaining 10 trials.

## Author Contributions

J.S., K.J.R. and G.G. designed the study, J.S. performed the experiment, J.S. and C.F. analyzed the data, all authors discussed the analyses, the results and wrote the paper.

## Supplementary Material

Supplementary Information Supplementary Information
