# Supplementary material for: Is ostension any more than attention?
Source: Sci Rep. 2014 Jun 16;4:5304. doi: 10.1038/srep05304 (PMC4058873; doi:10.1038/srep05304)
Supplement: Supplementary Information [file srep05304-s1.pdf]

Is ostension any more than attention?

Joanna Szufnarowska[1,*], Katharina J. Rohlfing[1], Christine Fawcett[2] and Gustaf Gredebäck[2]

[1]Emergentist Semantics Group, CITEC, Bielefeld University, Germany

[2]Department of Psychology, Uppsala University, Sweden

*Correspondence: jszufnarowska@techfak.uni-bielefeld.de

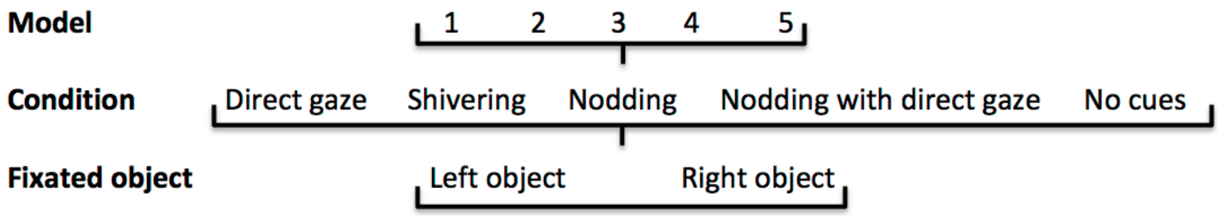

Supplementary Figure S1. **Schematic diagram of the corpus of recorded video clips.**
Each of the five models presented each of five conditions in two gaze directions (fixating at the object either on the left or right side of the screen in the last phase of the video). In total, the corpus of fifty videos was recorded. From that corpus, five versions of the stimuli were prepared. Since each version contained 20 videos, every recording was used twice.

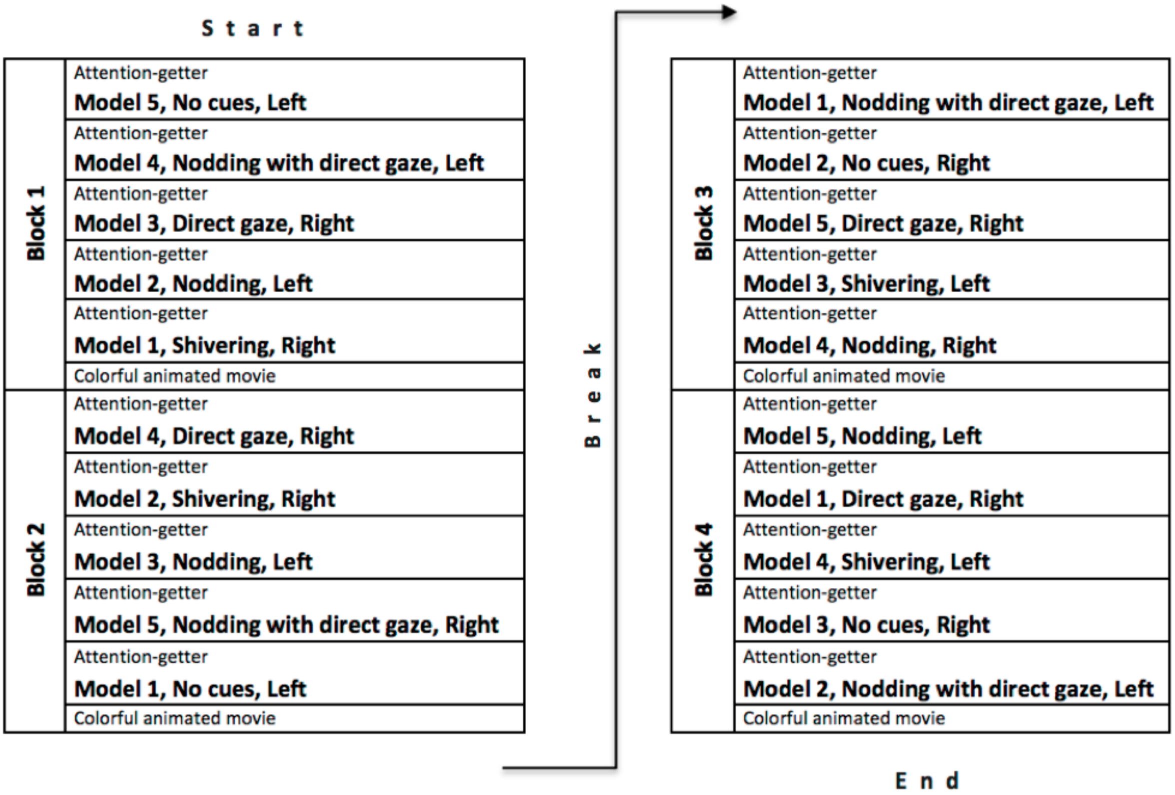

Supplementary Figure S2. **The order of the stimuli in one particular version of the experiment.**

Every time, after a brief attention-getter, the infant was presented with a trial with one of five female actors (e.g. **Model 5**), performing one of five conditions (e.g. **No cues**) and then looking at one of two objects: on right or left side of the screen (e.g. **Left**). Each version of the stimuli consisted of four blocks of five trials each. In sum, there were five versions of the stimuli. Each infant was presented with one of them.

Supplementary Results

Besides the difference score (DS) for the first head-to-object gaze shift, which is the standard assessment of infants' gaze following, two additional measurements were taken into account after the start of the actor's turn toward an object: the frequency of head-to-object gaze shifts and the duration of object fixation in the gazing phase. The DSs for each of them were calculated as they were for the first gaze shift. As such, the DSs above zero indicate that the infants made more eye movements from the model's head toward the attended object than toward the unattended one (the DS for frequency) and fixated longer on the attended object than at the unattended (the DS for duration). For each of the additional measurements, we again performed one-tailed single-sample t-tests for the measure of gaze following and one-tailed paired-sample t-tests for the comparisons between the No cues and each other condition.

For the No cues condition, neither the DS for the frequency of face-to-object gaze shifts nor the DS for the duration of object fixation significantly exceeded zero (frequency: DS = 0.02, $t(21)$ = 0.085, p = .467, d = 0.04; duration: DS = -0.10, $t(21)$= -0.546, p = .296, d = 0.24). Both the Direct gaze and the Shivering conditions resulted in significant DSs with large effect sizes for frequency of gaze shifts (Direct gaze: DS = 0.5, $t(21)$ = 3.328, p = .002, d = 1.45; Shivering: DS = 0.30, $t(21)$ = 1.944, p = .033, d = 0.85) and marginally significant DSs with medium effect sizes for the duration of gaze (Direct gaze: DS = 0.23, $t(21)$ = 1.377, p = .092, d = 0.60; Shivering: DS = 0.23, $t(21)$ = 1.497, p = .075, d = 0.65). Both Nodding and Nodding with direct gaze resulted in non-significant DSs and medium effects for the frequency of face-to-object shifts (Nodding: DS = 0.17, $t(21)$ = 1.166, p = .129, d = 0.51; Nodding with direct gaze: DS = 0.19, $t(21)$ = 1.315, p = .102, d = 0.58) and significant DSs and large effect sizes for the duration of object fixation (Nodding: DS = 0.51, $t(21)$ = 4.355, p <.001, d = 1.9; Nodding with direct gaze: DS

= 0.28, $t(21) = 1.907$, $p = .035$, $d = 0.83$).

Comparisons between No cues and each other condition for the frequency of face-to-object shifts revealed a marginally significant difference and medium effect for Direct gaze ($t(21) = -1.632$, $p = .059$, $d = 0.71$) and non-significant differences and small effects for the remaining conditions (Shivering: $t(21) = -0.938$, $p = .180$, $d = 0.41$; Nodding: $t(21) = -0.508$, $p = .309$, $d = 0.22$; Nodding with direct gaze: $t(21) = -0.615$, $p = .273$, $d = 0.27$). For the duration of object fixation, the difference from the No cues condition was non-significant with medium effect for Direct gaze (: $t(21) = -1.245$, $p = .114$, $d = 0.54$), marginally significant with medium effects for Shivering ($t(21) = 1.375$, $p = .092$, $d = 0.60$) and Nodding with direct gaze ($t(21) = -1.382$, $p = .091$, $d = 0.60$), and significant with large effect for Nodding ($t(21) = -2.523$, $p = .010$, $d = 1.10$).
